# Supplementary material for: Primary Health Care System Strengthening Project in Sri Lanka: Status and Challenges with Human Resources, Information Systems, Drugs and Laboratory Services
Source: Healthcare (Basel). 2022 Nov 10;10(11):2251. doi: 10.3390/healthcare10112251 (PMC9691080; doi:10.3390/healthcare10112251)
Supplement: Supplementary file 1 [file healthcare-10-02251-s001.zip › Annex_v1.pdf]

Annex-S1: Standard essential drug list to be available in PMCI

**Drug**
Adrenaline tartrate 0.1% injection 1ml Ampoule
Aspirin Tablet 150mg
Atenolol Tablet 50mg
Atorvastatin tablet 10mg
Beclomethasone diproprionate
Aerosol inhaler 50mcg metered dose
**200 dose unit MDI dry powder capsule for breath induced device**<br>100mcg DPI<br>200mcg DPI
**Chlorpheniramine malate**<br>Injection 10mg in 1ml Ampoule<br>tablet 4mg
Enalapril malate tab 5mg
**Frusemide**<br>injection 20mg in 2ml Ampoule<br>tablet 40mg
Glyceryl trinitrate tablet 0.5mg sublingual
Hydrochlorothiazide tablet 25mg
Hydrocortisone hemi succinate injection 100mg vial
Metformin tablet 500mg
Nifedipine slow release tablet 20mg
**Salbutamol**<br>Respiratory solution 0.5% in 10ml bottle<br>Tablet 2mg & 4mg
Theophylline slow released tablet 125mg

Annex-S2: List of laboratory services under basic laboratory package of PSSP

Category	Investigations
**Hematology**	Full Blood Count<br>Blood Grouping and Rh<br>ESR<br>Malarial Parasite<br>Blood Picture
**Biochemistry**	Lipid Profile<br>Total Cholesterol<br>Serum Creatinine<br>Blood Glucose<br>HbA1C<br>Oral Glucose Tolerance Test<br>Troponins (I/T)<br>ALT/AST CRP<br>Urine for bilirubin<br>Urine for glucose<br>Urine for ketone bodies<br>Urine for proteins<br>Urine for reducing substances<br>Urine for urobilinogen<br>Urine for β - HCG (qualitative)<br>Urine Microalbumin to Creatinine ratio
**Histopathology**	PAP-Smear
**Microscopy**	Urine/Stool Direct Microscopy<br>AFB stain for TB<br>Forward specimens for culture<br>Dengue Antigen and Antibody tests<br>VDRL/HIV (sample to be sent district STD Clinics)

Annex-S3: Facility assessment Checklist

Instructions for filling the form:

1. Please enter name of the person completing the questionnaire in uppercase letters

2. Always type/write inside the boxes or lines provided.

3. In case of numerical responses, always enter only one digit in each box ☐

Guide of the Interview:

1. Good day! My name is ______________________.

2. We are here on behalf of The International Union Against Tuberculosis and Lung Disease and Foundation for Health Promotion for conducting a survey of health facilities to assist the government in knowing more about health services in Sri Lanka.

3. Now I will read a statement explaining the study.

4. Your facility was selected to participate in this study. We will be asking you questions about various health services and empanelment of this PMCI under Primary Healthcare System Strengthening Project (PSSP) of Sri Lanka. Information about your facility may be used by the MOH, organizations supporting services in your facility, for planning service improvement or for conducting further studies of health services.

5. Neither your name nor that of any other health worker respondents participating in this study will be included in the dataset or in any report; however, there is a small chance that any of these respondents may be identified later. Still, we are asking for your help to ensure that the information we collect is accurate.

6. You may refuse to answer any question or choose to stop the interview at any time. However, we hope you will answer the questions, which will benefit the services you provide and the country.

7. If there are questions for which someone else is the most appropriate person to provide the information, we would appreciate if you introduce us to that person to help us collect that information.

8. At this point, do you have any questions about the study? Do I have your agreement to proceed?

Thank you for taking your time in completing the questionnaire/ checklist

A. General Information

A1	Name of the person completing the questionnaire (Assessor)	____________________
A2	Date of Assessment:	DD☐☐MM☐☐YYYY☐☐☐☐
A3	Name of the respondents	____________________
A4	Designation of the respondent/s	____________________
A5	Facility code:	☐ ☐ ☐ ☐ ☐
A6	Name of the facility	____________________
A7	Location of the facility	____________________
A8	District	____________________
A9	Province	____________________
A10	Type of PMCI (Please tick ✓)	1. Divisional Hospital-A ☐ 4. PMCU ☐<br>3. Divisional Hospital-B ☐<br>4. Divisional Hospital-C ☐
A11	Area (Please tick ✓)	1. Urban ☐ 2. Rural ☐
A12	Service hours	1. 24x7 ☐<br>2. Forenoon only ☐<br>3. Forenoon and afternoon ☐

A13	Remarks (if any)

B. Hospital Information Management System

B1	Availability of Computer (at least 1)?	1. Yes ☐	0. No ☐
B1a	Number of computers/laptops available for PMCI?	1. Desktop-<br>2. Laptops-	
B2	Availability of internet	1. Yes ☐	0. No ☐
B2a	If available, is it provided by the government or you use your private connection?	1. Government ☐<br>3. Both ☐	2. Private ☐
B3	Availability of functional electronic HIMS?? (Registration-PMCI/HLC)	1. Yes ☐	0. No ☐
B4	Availability of trained manpower to handle the HIMS?	1. Yes ☐	0. No ☐
B4a	Number of people trained to handle the HIMS? (document the designation)		
B5	Whether networking of local data server with national data center (cloud) is done	1. Yes, currently functional ☐<br>2. Yes, currently non functional ☐<br>0. No ☐	
B6	Is electronic database of individuals (with PHN) empaneled is available?	1. Yes ☐	0. No ☐
B6.i	If available, total people listed		

B6.ii	If not available, reason		
B7	Whether PHR printed in a hard paper board (printed booklet) available?	1. Yes ☐	0. No ☐
B7.i	Number of individuals registered with PHN in the facility		
B7.ii	Number of those registered who have received printed PHR booklet?		
B7.iii	Number of those registered who have PHR but not printed booklet (may be a different book or case record?		
B7.i	If printed PHR booklet not issued or partially issued, reason		
B8	Whether electronic PHR available?	1. Yes ☐	0. No ☐
B9	Whether PHR is updated during each visit patient makes?	1. Yes ☐	0. No ☐
B9.i	If no, reason		
B10	Whether the details of referral/ back referral are updated in PHR?	1. Yes ☐	0. No ☐
B11	Remarks (if any)		

C. Trained Human Resource in PMCI including associated HLC

	Designation	Number			
		Approved	Existing/ Available	Trained in PMCI empanelment (1 day training)	Trained on NCD guidelines (1 day training)
D1	Medical Officers				
D2	Nursing Officer (?PHNO)				
	Public Health Midwives				
	Dispenser				
D3	Pharmacist				
D4	Management Assistant				
D5	Health Promotion Officers				
D6	Counsellors				
D7	Dieticians				
D8	Instructors in physical education				
D9	Nutritionists				
D10	Dental surgeon				
D11	Attendants				
D12	Saukya Karya Sahayaka				
D13	Other healthcare staff				
D14	Public Health Laboratory Technician				
D13	Remarks (If any)- (Especially add details here on if the existing is ‘0’, how the work is managed?				

D. Equipment

Sl.No	Equipment	Number				
		Sanctioned	Available	Functional/ Calibrated (C)	Approximate number of days not functional in 2020	Number of test strips consumed in 2020
E1.i	Glucometer					
E1.ii	Cholesterol meter					
E1.iii	BP apparatus			C-		
E1.iv	Stethoscopes					
E1.v	Weight scale			C-		
E1.vi	Height measuring rod / Board					
E1.vii	Weighing scales for infants			C-		
E1.ix	Measuring tape					
E1.x	Ophthalmoscope					
E1.xi	Snellen chart					
E1.xii	Tuning Fork					
E1.xiii	Examination bed					
E1.xiv	Tongue depressors					

E1.xv	Torch				
E1.xvi	Tendon hammer				
E1.xvii	Thermometers (digital)				
E1.xviii	Nebulizer				

E. Laboratory Services through PMCI

Sl.No	Laboratory Tests	Available (Y/N)	If available: PMCI / Public-Cluster / Private-Cluster / Others	If not PMCI, sample collection and transport available (Y/N)	Number of tests done in 2020	Approximate number of days the investigation was not available in 2020
F1	Blood Glucose					
F2	HbA1C					
F3	Total Cholesterol					
F4	Lipid Profile					
F5	Serum Creatinine					
F6	Urine for glucose					
F7	Urine for ketone bodies					
F8	Urine Albumin to Creatinine ratio					
F9	Oral Glucose Tolerance Test					
F10	PAP-Smear					
F11	ALT/AST					
F12	Complete Blood Count					
F13	AFB stain for TB					
F14	HIV					
F15	Blood Grouping and Rh					
F16	Dengue Antigen or Antibody tests					
F17	Malarial Parasite					

F18	Remarks (if any)

F. Essential NCD Drugs and Supply Chain

Sl.No G1	Drugs	Available (Y/N)	Quantity in last year (2020)				Any stock out last year? (Y/N)- If Y, Duration??	Closing stock last month	Date of last indent	Date of receipt
			Indented	Received	Disbursed	Expired				
1	Adrenaline tartrate (0.1%)- 1ml Ampoule									
2	Aspirin tablet 150 mg									
3	Atenolol tablet 50mg									
4	Atorvastatin tablet 10mg									
5	Enalapril maleate tablet 5mg									
6	Frusemide – Injection 20mg in 2ml Ampoule									
7	Frusemide tablet 40mg									
8	Gliclazide tablet 40mg and 80mg									
9	Glyceryl trinitrate tablet 0.5mg sublingual									
10	Hydrochlorothiazide tablet 25mg									
11	Losartan tablet 50mg									
12	Metformin tablet 500mg									
13	Nifedipine slow release tablet 20mg									
14	Salbutamol Respiratory solution 0.5% in 10ml									

15	Salbutamol 2mg tablet									
16	Salbutamol 4mg tablet									
17	Theophylline Slow released tablet 125mg									

G2	Drug storage facility adequate?	1. Yes ☐ 0. No ☐
G3	NCD drugs are dispensed in blister packs?	1. Yes, all drugs ☐ 0. No ☐<br>2. Yes, some drugs (partial) ☐
G3.i	If not available or partial, reason	
G4	NCD drugs are dispensed for one month or more than one month?	1. Yes ☐ 0. No ☐
G4.i	If not dispensed for a month or dispensed for more than one month, reason	
G5	Availability of trained manpower to handle the drug supply chain	1. Yes ☐ 0. No ☐
	If Pharmacist and/or dispenser are not available, who is dispensing medicines?	
G6	Whether online drug indenting system is available?	1. Yes, availabe and Functional ☐ 0. No ☐<br>2. Yes, available but not functional ☐
G7	Remarks (if any)	

H. Friends of Facility and Grievance Redressal

H1	Is "Friends of Facility" committee established in PMCI?	1. Yes ☐ 0. No ☐
H2	Number of Members in Friends of Facility committee	
H3	Whether 'Friends of Facility' has at least one member from each GN	1. Yes ☐ 0. No ☐
H4	Number of 'Friends of Facility' meetings conducted in 2020	
H5	Number of meetings for which minutes have been documented in 2020	
H6	Number of patient grievances discussed/addressed during Friends of Facility meeting in 2020	
H7	Date of last Friends of Facility meeting	
H8	Number of members who attended the last meeting?	
H9	Is there a box or mechanism for dropping the patient grievances?	
H10	If available, how many grievances were submitted during 2020	
H10.i	Of those submitted, how many were discussed in Friends of Facility meeting	
H10.ii	Of those submitted, how many have been addressed	
H11	Remarks (if any)	

H. Infrastructure

I1	Drinking water	1. Present ☐ 0. Absent ☐
I2	Electricity	1. Present ☐ 0. Absent ☐
I2i	Electricity backup	1. Present ☐ 0. Absent ☐
I3	Waiting area with reception counter	1. Present ☐ 0. Absent ☐
I3.i	Number of seats in waiting area	
I3.ii	Average number of OPD attendance in last one month	
I4	Bill board displayed (outside)	1. Present, in all three language ☐<br>2. Present but not in all three ☐ language<br>0. Absent ☐
I5	Map of empaneled area displayed	1. Present ☐ 0. Absent ☐
I6	OPD label	1. Present, in all three language ☐<br>2. Present but not in all three ☐ language<br>0. Absent ☐
I7	Signboards	1. Present ☐ 0. Absent ☐
I8	Notice board with services available	1. Present, in all three language ☐<br>2. Present but not in all three ☐ language<br>0. Absent ☐
I9	Notice board with empanelment status	1. Present, in all three language ☐<br>2. Present but not in all three ☐ language<br>0. Absent ☐
I10	General office (room 1)	1. Present ☐ 0. Absent ☐
I11	Consultation room 1	1. Present ☐ 0. Absent ☐
I12	Consultation room 2	1. Present ☐ 0. Absent ☐
I13	Consultation room 3	1. Present ☐ 0. Absent ☐

I14	Dispensary / dispenser room	1. Present ☐	0. Absent ☐
I15	Drug store/ supplementation store (with air conditioner)	1. Present ☐	0. Absent ☐
I16	Laboratory room (with sink)	1. Present ☐	0. Absent ☐
I17	Medical officers rest room with bathroom	1. Present ☐	0. Absent ☐
I18	Rest room for other staff with bathroom	1. Present ☐	0. Absent ☐
I19	Minor staff room with bathroom	1. Present ☐	0. Absent ☐
I20	Overseer or matron room 1	1. Present ☐	0. Absent ☐
I21	Overseer or matron room 2	1. Present ☐	0. Absent ☐
I22	Patient toilet	1. Present ☐	0. Absent ☐
I22.1	Number of toilets for male /number which are disability friendly		
I22.ii	Number of toilets for female / number which are disability friendly		
I23	Meeting room with pantry	1. Present ☐	0. Absent ☐
I24	General store	1. Present ☐	0. Absent ☐
I25	Instrument room	1. Present ☐	0. Absent ☐
I26	Health promotion room or health education room	1. Present ☐	0. Absent ☐
I27	Dental room	1. Present ☐	0. Absent ☐
I28	ETU room or space for emergency care services	1. Present ☐	0. Absent ☐
I29	Dressing room	1. Present ☐	0. Absent ☐
I30	Vaccination room/ injection room	1. Present ☐	0. Absent ☐
I31	Office space for PHMs	1. Present ☐	0. Absent ☐
I32	Office space for PHIs	1. Present ☐	0. Absent ☐

I33	Quarters for MO (1)	1. Present ☐	0. Absent ☐
I34	Quarters for MO (2)	1. Present ☐	0. Absent ☐
I35	Quarters for PHMs	1. Present ☐	0. Absent ☐
I36	Audiovisual equipment's for Health Education (Details if present)	1. Present ☐	0. Absent ☐
I37	IEC- Leaflets	1. Present, in all three language ☐<br>2. Present but not in all three ☐ language<br>0. Absent ☐	
I38	Quarters for nurses	1. Present ☐	0. Absent ☐
I34	Remarks (if any)		

Annex-S4: Interview guide for healthcare providers

Respondents: Program Manager/ Doctor / nursing staff/ laboratory technician

AIM 1	Objective
To assess if the PMCIs and their services have been reorganised in line with the standards and clinical care protocol endorsed by the Ministry of Health (MOH) and explore the challenges perceived by the healthcare providers in the reorganisation process.	Explore the challenges with retention and training of health staff of PMCI as perceived by the programme managers and health staff

Greetings	
Can you please give us an introduction about yourself?	Probe for his/her tenure of service over here as the treating physician/ healthcare staff/ program officer, previous experience, training, if any, in relation to on NCD management
What is your current role and how do you plan to deliver your responsibilities?	Probe for daily routine, work plan, common issues faced
in your opinion, whether the PMCI have adequate number of human resources as recommended in the PMCI guidelines	Probe for challenges in retaining medical officers, nursing staffs, lab technicians and other staff, frequency of staff transfer, if any, and issues in staff replacement
Whether you have been trained on the reorganized PMCI or PSSP project?	Probe for ease of understanding in during the training, duration of training, applicability of the training in day to day practice under the PSSP project at the PMCI, challenges related to training, if any. Whether any materials was given, post training support and clarification of queries.
What kind of challenges you are facing while working together with other staffs at the PMCI	Probe for delaying of work, incomplete or inadequate training, any discussion meeting happened at the PMCI to understand regarding the PSSP project, any other challenges in PMCI functioning under the PSSP project.
What kind of challenges you are facing while providing healthcare to patient the PMCI	Probe for delaying of work, difficulty in understating due to incomplete or inadequate training, any other challenges in PMCI functioning under the PSSP project.
What kind of challenges in coordinating with GNs and apex hospitals?	Probe for referral system functioning, follow-up of patients after back referral, challenges in coordination with staffs from GN and apex hospital, if any.

AIM 1	Objective
To assess if the PMCIs and their services have been reorganised in line with the standards and clinical care protocol endorsed by the Ministry of Health (MOH) and explore the challenges perceived by the healthcare providers in the reorganisation process.	Explore the challenges in supply chain management using MSMIS and reasons for 'urgent' or local purchases as perceived by the programme managers and health staff
In your opinion how was the overall ease of operation of MSMIS?	Probe for the process of MSMIS, availability of trained manpower, a functional computer with internet connectivity, other challenges related to operating the MSMIS
What kind of challenges you faced while indenting the essential drugs for NCD management	Probe for availability of all essential drugs of NCD, ease of changing the requirement during every indenting, options of adding any new drug/ dosage, procedure the required essential drug is not available in the central store at the time of indenting, challenges in receiving the non-available drugs
What is the average lead time from the generation of indent and receiving the drugs at the PMCI	Probe for different lead time for different drugs or all drug will be delivered at the same time only
What kind of challenges did your PMCI faced for maintaining buffer stock all essential drugs?	Probe for knowledge on buffer stock, calculating and indenting for buffer stock, receiving buffer stock
In your opinion in what situations or for what drugs local purchase or urgent purchase was made?	Probe for issues in indenting from PMCI or central store, management of fund for local purchase, what all drugs and for what duration this local purchase was made
What was the inventory control system managed in the PMCI	Probe for the inventory control process followed at the hospital, the healthcare staff who is managing and the frequency of monitoring by the head of PMCI
Can you please tell how the near to expiry drugs were managed	Probe for expired drugs and near-to expiry date drugs present in last one year, what is the mechanism for management of the same?
What is the kind of training provided for supply chain management?	Probe for special training on MSMIS, supply chain management and inventory control, the individual who attended it from PMCI, or any other support is provided for supply chain and inventory control?

AIM 1	Objective 5
To assess if the PMCIs and their services have been reorganised in line with the standards and clinical care protocol endorsed by the Ministry of Health (MOH) and explore the challenges perceived by the healthcare providers in the reorganisation process.	Understand the mechanisms (patient referral or sample collection and transport) established for laboratory investigations and challenges associated with it, as perceived by the programme managers, health staff of PMCI and the patients

What kind of challenges were faced while providing laboratory services	Probe for the process of laboratory investigations, tests at PMCI, private facility test, patient feedback regarding the laboratory tests
Can you please tell whether all the essential tests for NCD management is available at the PMCI	Probe for the process of laboratory investigations at the PMCI, challenges in sample collection, biomedical waste management, process of non-available tests at the PMCI
What kind of challenges you faced in sample collection and transporting of the samples at the PMCI?	Probe for challenges in sample collection, transporting, vehicle, cold storage, pre-processing of the sample.
In your opinion, how quickly the results were obtained	Probe for duration of waiting time after sample collection and provision of results to the patient, mechanism for sending the results back to the PMCI, process of coordination between public and private sector for providing the lab services
Whether the trained man power was available in the PMCI in the last one year?	Probe for adequate manpower to provide lab services and skills of the laboratory technician
Whether there was any disruption of any of the lab services in last one year and can you please tell what was the reason?	Probe for reasons such as equipment related problem, consumables related, manpower related, any other etc
