# Supplementary material for: Primary Health Care System Strengthening Project in Sri Lanka: Status and Challenges with Human Resources, Information Systems, Drugs and Laboratory Services
Source: Healthcare (Basel). 2022 Nov 10;10(11):2251. doi: 10.3390/healthcare10112251 (PMC9691080; doi:10.3390/healthcare10112251)
Supplement: Supplementary file 1 [file healthcare-10-02251-s001.zip › Suplementary_Table_v2.pdf]

Supplementary Table S1: Qualitative data on challenges with human resource management and training in PMCIs supported by PSSP in Sri Lanka, 2021

Sub-theme/ Category	Description	Quote(s)
Pre-existing issues in HR management	These are the chronic challenges related to trained manpower which existed in PMCIs even prior to the implementation of PSSP. However, some of these challenges might have come to notice or worsened due to PSSP which required additional human resources for adequate implementation.	
Shortage of staff	The HCWs felt that the PMCIs are usually understaffed with shortages of doctors, nursing officers, public health nurses, and data entry operators in the PMCIs. Also, the HCWs opined that there is a gross shortage of data entry operators and public health nurses, who are necessary to carry out the activities related to PSSP.<br><br>According to HCWs, the reasons for inadequate staff in the PMCIs are: a) Lack of recognition for performance at PMCI, b) Reluctance of HCW to work in PMCI and c) Frequent turnover of trained HCWs from the PMCI	One of the `programme managers referring to the human resources situation in the province said,<br><br>*"There is a problem with HR. This (PSSP) is a huge task. Things they need to do in achieving targets are out of their routine work. So, HR should be increased. Anyway, there is a deficiency of HR in our system, especially at the peripheral level. So that affects PSSP"*
Reluctance of HCW to work in PMCI	According to HCWs, there is unwillingness among their colleagues to work in the peripheral PMCIs considering their preference to stay in urban neighbourhoods. Even on getting posted to PMCI, the HCWs don't report to their duties and find excuses to avoid such a posting.	A medical officer referring to deficiency of doctors said,<br><br>*"You know some people don't like this system. Like when a doctor is being transferred to a central dispensary (PMCI), they always try to go off. They never like to report to that place and start their duty. They're scared of coming to a place like this (rural) and also they're not very happy"*
Lack of recognition for performance	The HCWs feel that there is lack of recognition for the efforts they put at PMCIs. Despite excellent performances from	One medical officer in-charge referring to human resources deficiency and support from the superiors said,

	HCWs, the provisional/district level programme managers fail to recognise and appreciate them.	*"I suggested to the MO-Planning to come to the PMCI and give them (field staff) certificates. The answer given was very disappointing even to mention. The answer was 'we don't have funds".*"
Frequent turnover of trained HCWs from the PMCI	The HCWs alleged that temporary recruitment and a transfer policy with frequent turnover of the trained staff is leading to deficiency in functioning of the PMCIs. Also, they feel that annual transfer of doctors and requested transfers of other staff harms the continuity of care in the facilities. The temporary recruitment of data entry operators has led to frequent turnovers as the recruited staff resign on getting a permanent government job.	A programme manager speaking about frequent transfers of staff said,<br>*"Yes, it's an issue. Most of the time we train the nurse who is assigned to HLC (healthy life style center) and give her the responsibility of PSSP registration. So, if the nurse has been transferred to another hospital, the new one takes time to get oriented. There are motivated and dedicated people in certain places. If they are transferred, that affects the process*."<br>A nursing officer speaking about the challenges with data entry operators (DO) mentioned,<br>*"But now there is a problem with permanent DO position. These days there are temporary DOs appointed frequently through the government job offering system. However, they are shifting on getting other permanent positions. So, it is a problem."*
Challenges with introduction of PSSP	The HCWs feel that the implementation of the PSSP in PMCIs with depleted healthcare staff has brought in more challenges. Also, the existing HCWs were not able to execute the activities of the PSSP.	
Deficiency in training on PSSP	The HCWs reported that there was deficiency in the training due to lack of adequate funds and resource persons for training, lack of standardised training content, limited	

	exposure to practical aspects during training and inability of HCWs to attend training	
Lack of adequate funds and resource person	The HCWs expressed that there is lack of adequate and experienced resource persons for leading the training under PSSP. They felt that this was one of the reasons for cancelling the trainings along with the existing COVID-19 situation. The programme managers opined that there is a lack of resource allocation for training under PSSP compared to other development projects implemented in Sri Lanka.	A programme manager said, *"The current COVID pandemic became a big challenge. We also get difficulties in finding adequate resource persons."* A programme manager said, *"…In PSSP, only a limited amount is allocated for trainings"*
Lack of standardised training content	The HCWs felt that there was no standardised content for trainings from various units supporting or monitoring the activities at PMCIs. Lack of such consistency led to confusion in carrying out the activities in the PMCIs.	A nursing officer said, *"The training was conducted by the NCD and the PSSP. The question most of us had was the NCD and the PSSP (during training) made totally different statements. By the NCD staff, we were asked to register any patient from any part of the island and give medicine if they don't have an infectious disease. The PSSP stated this differently (to include only patients from the GN division)"*
Limited exposure to practical aspects	The HCWs complained that during the trainings, the practical issues for carrying out activities in the PMCIs and the catchment area were not discussed well. Not addressing the practical issues during the training led to challenges in implementation of PSSP.	A programme manager said, *"I was given (training). But I need to gain practical knowledge. That had not been given adequately."*
Inability of HCWs to attend training	The HCWs expressed that they were not able to participate in some of the PSSP trainings due to lack of replacement to attend work at PMCI and restrictions from superiors to attend trainings.	A medical officer in a single doctor station said, *"When PSSP calls me for meetings, and when I inform the MO planning that I have a meeting. Sir scolded me not to go for meetings in Colombo, saying that 'The center will have to*

	With the existing shortfall of staff, the HCWs could not attend the meeting as that meant closing the services altogether at the PMCIs. When they had to choose between delivery of services and trainings, the programme managers suggested choosing the former over the latter to ensure uninterrupted service delivery at PMCI.<br><br>The HCWs at PMCI also felt that there is some restriction from the superiors (programme managers) to permit them to attend the meeting/training of PSSP. Also, some of the travel charges are not reimbursed and study leave is not granted for such trainings.	*close, who is going to see the patients?', this is how he spoke to me. I expected him to say, if you are going for the meeting arrange something"*<br><br>A medical officer quoted an incidence on such restriction and how it was negated,<br><br>*"I forwarded the email I received to Sir. He said that he doesn't have time to go through the email and that I need not go. I don't know whether you will believe me, I put in a sick leave and came for the meeting. I travelled by bus to Colombo"*
Additional responsibilities to the existing staff	The PSSP required mobilization of the individuals from the community for empanelment and online registration with entry of demographic and screening details. However, according to HCWs, there was no public health nurse officer (PHNO) for carrying out community activities and the data entry operator to manage the online registration. Also, with the introduction of sample collection and transportation, the nursing officers had to be available for sample collection. However, without additional recruitment for these activities, the work was redistributed among the existing staff in the PMCIs. According to HCWs, the additional workload with PSSP has led to stress, exhaustion and inability to take leave.	A nursing officer involved in empanelment and data entry for PSSP said,<br><br>"I have to do all the work related to nursing care. Furthermore, I have to do the work of this newly started PSSP project work too."
Non-availability of leave	As the staff are multi-tasking with PSSP and don't have replacements, they find it very difficult to avail leave.	One of the nursing staff said,<br><br>*"Sometimes, if one (nursing officer) takes leave and does not come to work, there will be no one coming for cover-up. So, we have to look after these cover-ups ourselves too.*

		Furthermore, since there is no one to cover-up, it's a very hard thing to get a leave even for drug dispensers. Therefore, taking leave is a very stressful thing. We, both (nursing officer) cannot have a leave at the same time."
Exhaustion of HCWs	The HCWs feel exhausted with additional work because of PSSP.	A medical officer in-charge went on say,<br>*"Truly speaking, I think like we are a bit exhausted with this PSSP project.* Now if I can get a medical officer instead of the RMO, I mean that would be a lot of help. And even for the nursing officer, if she can have a helping hand that is so good."
Work stress among HCW	The HCWs feel that the additional work they have with the project is making them feel stressed.	A medical officer managing the data entry under PSSP went on to say:<br>*"I can see that the person (data entry operator) who has to come doesn't come and the data entry operator in service is removed. This is a draw back and stressful."*<br>The medical officer went on to describe the multi-tasking by the staff at PMCI,<br>*"In the morning I treat the patients, handle the clinics, issue drugs and enter the log books. Go to get medicine, I use my vehicle to bring the medicine. Then I enter data in the books. Then there are a lot of books and admission cards to be entered and I take them home and complete the work. Actually, I was unable to have proper sleep."*

Supplementary Table S2: Qualitative data on challenges in drug stock management in PMCIs supported by PSSP PSSP in Sri Lanka, 2021

Sub-theme/ Category	Description	Quote
Inadequate storage facility	The HCWs complained that there were lacunae in the drug storage facility of the PMCIs. The three deficiencies highlighted by the HCWs were insufficient drug storage space, lack of air conditioning (AC) in drug stores and storage of drugs in transparent pill bottles.	
Insufficient drug storage space	The HCWs felt there is shortage of space for storing the drugs and thus, the facilities are not able to maintain the buffer stock for the drugs.	A pharmacist while providing the reason for not maintaining adequate buffer stock, said<br>*"We have the space problem, due to which, we can't keep it (buffer stock) too"*<br>A medical officer while explaining the challenges faced by them without having dedicated space for storing drugs, said<br>*"The drugs were attacked by rats and even the plastic chairs that the patients sit on. We cannot issue drugs that have been damaged by rats"*
Drug storage in transparent bottles	The HCWs mentioned that the transparent pill bottles are used to store the loose drugs as the amber coloured bottles are not available. They feel such use of transparent pill bottles can harm the quality of the drugs stored in the facility.	A medical officer while describing the lacunae in the drug storage, said<br>*"I officially requested a glass cabinet to keep the drugs.... It is difficult to store the drugs because the transparency of the glass."*
No AC in drug stores	The HCWs feel there is need for several improvements in drug storage, of which the installation of AC is the major one. They feel that the high temperature can damage the quality of the loose drugs stored in the facility.	A medical officer said,<br>*"Actually, my drug store needs a lot of improvements. It's an old one. It is not air conditioned. Though there are cupboards they are old. I don't have a proper table. Lack of AC is a*

		significant issue. I think the temperature goes above 25 Celsius and that affects the quality of drugs."
Lack of blister packs and dispensing of loose drugs	Though PSSP intended to deliver the drugs in blister packs, according to HCWs, the blister packs were not available for most of the drugs in the PMCIs (except for Aspirin). The HCWs felt that the loose NCD drugs given for a month and stored in the plastic covers may be of poor quality.	A medical officer talking about the blister packs said, *"We have to give the drugs in loose form and when we give the medicine for a month we request the patients to place the tablets in a bottle, because by the end of the month the quality of the tablets can vary"*
Concerns on quality of loose drugs	The HCWs complained that the drugs are still not available in the blister packs and the loose drugs are perceived to be of low quality among the general public. The HCWs felt that there is high chance of contamination while handling the loose drugs, as the pills are counted with bare hands. This is a challenge as the pill counter machine is not available in all the facilities.	A medical officer talking about loose drugs said, *""Yeah, now what matters is the quality. For example, when I give thyroxine or metformin, it is all as loose drugs. So, the quality matters"* A dispenser describing the challenges with the loose drugs, said *"We get medicine mostly in loose quantity. At the end of the month, stocks have to be taken and it is difficult to count each tablet and assess the balance amount and those that might be liable for contamination"*
Unavailability of drugs leading OOP purchase	According to HCWs, all the drugs are not readily available in the PMCIs and the patients have to purchase them from the private pharmacy leading to out-of-pocket expenditure. There are shortages of both essential drugs and those not in the essential drug list, however, prescribed at the PMCIs. The HCWs felt the common reasons for such shortages are suboptimal drug supply chain management and maintenance of low buffer stock.	

Shortage of essential drugs	The HCWs mentioned that though things have improved with PSSP, still there are rare instance of shortages of essential drugs in the PMCIs. Notably, there is a shortage of drugs used for management of NCDs.	A medical officer talking about the essential drugs, said<br>*"Yeah, we sometimes have shortages. In 2020 we didn't have any issues with shortages, but then again in 2021 we had shortages here. Some other areas also had issues with glycoside and Losartan."*
Suboptimal supply chain management	According to HCWs, the shortage of drugs is due to sub-optimal drug supply chain management because of challenges with Medical Supplies Management Information System (MSMIS), delivery of drugs and drug forecasting.	
1) Medical Supplies Management Information System (MSMIS) related	The MSMIS is recommended to be established in the PMCIs for supply chain management of drugs. However, the HCWs feel that the system is not well established in all the facilities. Also, when introduced, there was an issue with handling of the MSMIS due to inadequate training of the key personnel handling it.	
a. Non-availability of MSMIS	There are PMCIs where the MSMIS has not yet been introduced.	The medical officer from PMCI with no MSMIS, said<br>*"In the base hospitals there is a system where you can order drugs online, but we don't have… Our hospital was selected and they (biomedical unit) took the details to fix the necessary parts to the hospital. Then they came and fixed them (system for MSMIS) near the dispensary, but did not work"*
b. Inadequate training	The pharmacist and dispensers using the MSMIS felt that they are not well trained to use the MSMIS for indenting.	A pharmacist managing the MSMIS talking about the ease of using the system and training said<br>*"I didn't participate for the system training (due to leave). So, I do it by asking from others. So, there is an issue. I had to face*

		some issues while I am working. So, it is better to have the training again."<br>A dispenser involved in supply chain management of drugs, said<br>*"I haven't got any training on supply management (MSMIS)."*
1. Delivery of drugs	The HCWs mentioned that there are challenges with delivery of indented drugs. There was no transportation facility for getting the drugs from the drug stores and the delivery of indented drugs was delayed.	
a. No transportation for drug delivery	The drugs are usually transported from the drug stores at RMSD or MSD to PMCIs. The HCWs mentioned that facilities for transportation of drugs from stores to PMCI are not always available requiring use of the personal vehicle of the staff to carry the drugs.	A medical officer said,<br>*"They (RMSD) sent it in a vehicle, not always. Most of the time I go in my vehicle, bend forward the seat and bring the boxes of drugs."*
b. Delay in receipt of indented drugs	The dispensers and pharmacist mentioned that even with the new system (MSMIS), there are delays in receipt of the indented drugs.	A pharmacist explaining the process and delay in the supply of drugs, said<br>*"This fact is really important. The estimation is not received on time. We send an annual estimation. It will be prepared at the end of August or beginning of the September. It should be received by January, but normally we received it February or March. So roughly it takes two months to reach here."*
Lack of trained pharmacist for forecasting	The programme managers complained that the main reason for the issues with the supply chain was due to the lack of a trained pharmacist for sharing the forecast in a timely manner.	A programme manager describing the deficiencies in forecasting said,<br>*"Actually at DH (divisional hospital) level, pharmacists are employed according to cluster basis. We have dispensers for*

		each institute. But one pharmacist looks after few DH's. That happens due to lack of human resources"
Low buffer stock	The HCWs feel they maintain the low buffer stock to avoid challenges in redistribution when in excess and also to accommodate the drugs in the available storage space. This sometimes leads to shortages of drugs when the supply of drugs is delayed.	The dispenser explaining the reason for not having buffer stock of three months, said<br>*"That means, we make the estimates to have a buffer stock. So as not to have shortages of drugs, what we do is, when we calculate the estimates, even for a year, we calculate for one month extra"*
Unavailability of non-essential drugs	According the HCWs, with increased utilization of the care at PMCIs, there are patients who require some class of drugs over and above those listed in the essential drug list. In most instances when patients require the drugs not in essential list, they had to purchase them from the private pharmacy. The prescription of non-essential drugs was increasing as the patients were being referred back from the secondary and tertiary hospitals to PMCIs for care and follow up.	A programme manager explaining the challenges with drugs for those referred from higher centres to PMCI, said<br>*"A PMCI couldn't receive this level of drugs, only specialized and teaching hospitals can receive this level. This issue is there, when back referrals are made to peripheral hospitals, most patients come again here, what is the reason; this drug is not available there, especially cardiology, neurology, anti-hypertensive, there are new drugs… generally they have (says a list of commonly available drugs), but some new drugs, in anti-cholesterol (says two kinds of drugs), then in cardiology, most of the drugs used in ischemic heart diseases are not available in peripheral hospitals"*
No local purchase drugs	According to HCWs, the PMCIs were not allowed to make local purchases of drugs which are not available in the facility. This is especially true for drugs which are not in the essential list – these are neither supplied by RMSD nor are there any options for local purchase. Even if they want to	A medical officer talking about the issues with drugs, said<br>*"Problem is local purchasing is not practiced here. It should be implemented."*<br>Another medical officer said,

	do the local purchase, there is lack of monetary resources for the same in PMCIs.	*"We don't do local purchases. We have a very limited petty cash. It's just 5000 LKR."*
Long process for procurement	The HCWs feel, though, there is a way to procure the drugs not in the essential list, the process is lengthy and requires the request to be routed through the consultants (specialist doctor). Not all the PMCIs have the consultants conducting the specialist clinics and this process may not be feasible.	A pharmacist describing the process for procurement of drugs not in the essential list, said *"As we have a consultant, we have sent a special request regarding those drugs. Because DH is managed by the RMSD, once we give our request they send the request to the MSD but not as our request. So most of the time they link our requests with base hospitals and send to the MSD… This procedure takes nearly one month."*

Supplementary Table S3: Qualitative data on challenges in laboratory investigations conducted through PMCI supported by PSSP in Sri Lanka, 2021

**Sub-theme/ Category**	**Description**	**Quote**
Non-availability of investigations at PMCI	The HCWs mentioned that in most of the PMCIs, except for blood glucose and cholesterol estimation using point of care devices, the other investigations are not available within the facility.	A medical officer describing the situation said<br>*“And when it comes to NCD related investigations, we don't have facilities to do lipid profile, HBA1C, micro-albumin… you know micro-albumin is very important to assess kidney function, but we don't have a facility for that”*
Lack of space for expanding laboratory	The inherent problem in the PMCIs that the HCWs mentioned is lack of space for expanding the laboratory services.	A medical officer mentioning the same said,<br>*“The lab space is not enough. We hope to expand it and for the moment the basic investigations are carried out… The major problem is that, we don't have enough space in the lab. All the items are packed inside the lab”*
Lack of auto analyser	The HCWs mentioned that there are no auto analysers for processing the samples. Even when the auto analysers are present, they are not installed or serviced. Though there has been funding support from the PSSP for procuring the auto analysers, they are yet to be procured.	A medical officer highlighting the deficiency of the auto analyser in the facility, said<br>*“Also, we got a three-part analyser as a donation. But the company has changed and the new company said that they will take it back. As the company changed, there is a problem in servicing the machine as well. Therefore, we will lose an analyser.”*<br>A programme manager confirming that auto analysers will be made available at PMCI in the near future said,<br>*“About laboratory equipment, we have PSSP funding for that, we have planned and we will receive those in future. The analyser will be received in the future.”*

Inability to get investigations done in cluster through SCT within cluster	As not all the investigations were available in the PMCI, the PSSP recommended establishing the laboratory network within the cluster with sample collection at PMCI and transportation to an apex laboratory for processing. Some of the challenges that the HCWs mentioned about sample collection and transportation for investigation are described below.	
Not initiated or lack of support for SCT	Though the PSSP proposed sample collection and transportation, the HCWs complain that the system is not yet established in some of the PMCIs. They also mentioned that there is lack support for setting up the system.<br><br>The programme managers feel that there are some deficiencies in personnel and laboratory equipment to establish such systems immediately. They feel that it will take some more time and might require additional resources to set up such a system.	A medical officer complaining about no support for establishing SCT, said<br><br>*"Then XXX sir (programme manager) said to me personally, that I have to do primary care and not tertiary care; so that he will not grant me permission to send the samples through the minor staff member to XXX hospital."*<br><br>A programme manager acknowledging the fact that the laboratory network is not functional, said<br><br>*"There are some institutions where the requirements are not fulfilled. We have planned to implement a laboratory network in our district and this will be completed by 2023."*
Shortage of staff for sample collection	The HCWs reported that due to shortages of staff and not having dedicated laboratory technicians, it becomes difficult to provide sample collection services at PMCI.	A nursing officer explaining the same, said<br><br>*"There is a problem like this- If I take a leave, there will be no one to collect samples. I live close to this place and even can see this place from home. There are days I used to come and draw the blood in the morning while I'm on my leave…. So, I finish the work here quickly on my leave days."*

Inadequate personnel for sample transportation	In some of the facilities, the HCWs reported that there are no dedicated personnel for transportation of the samples from PMCI to laboratories in secondary or tertiary hospitals. In such cases, the sample is collected at PMCI and the patient carries it to laboratory.<br>Even in facilities where there is support for sample transportation, the HCWs reported that only one person is responsible for transportation of samples from the whole of the cluster. When the courier person is on leave, the sample collection and transportation is not possible.	A medical officer of one such facility, said<br>*"What we do here is punch and take the blood and test on the glucometer. The nurse draws the blood of the patient and he or she takes it to a lab"*<br>A nursing officer in-charge of sample collection, said<br>*"And we have only one person as a courier person, for the whole cluster. So he also has the same issue. He can't take leave."*
Long turnaround time	The HCWs mentioned the delay in receipt of reports as one of the problems with the sample collection and transportation. It will take a minimum 24 hours to receive the report. Also, with COVID-19 and transportation of samples once in two days, it is taking a minimum of 48 hours for receipt of test results.	A nursing officer mentioned,<br>*"If we consider the timing of test reports, we draw the blood sample at 8 am and results will be available only at 10 am the following day at the time the courier person comes to collect the samples of that day. Therefore, if we can get test results done a little earlier than this, It would be better… the delay of reports now is two days (with COVID-19 situation the samples are transported once in two days)"*
Unavailability of laboratory technician	The HCWs feel that the unavailability of trained laboratory technicians is one of the major challenges in ensuring laboratory investigations through PMCI. The laboratory technicians are not available even at the apex laboratories leading to failure of sample collection and transportation.	A programme manager acknowledging the deficiency in trained laboratory technicians, said<br>*"We have a shortage of 20 for the XXX district only. We function without MLT's (medical laboratory technicians) and run on a relief basis."*<br>A programme manager from another province also said,

		"There is a problem regarding that (availability of investigations within the cluster), due lack of MLT. It will be done by 2023"
Insufficient supply of reagents/consumables	According to HCWs, the shortage of reagents occurs even in the apex laboratories leading to the cancellation of certain investigations.<br><br>The other issue highlighted by the HCWs was the lack of consumables (strips) for utilizing the available equipment. The issue worsened with introduction of PSSP as the screening utilization increased in each of the facilities.	A medical officer mentioning the shortage of reagents in PMCI, said<br><br>*"Also, recently we got a biochemistry analyser. We have installed it. But still we didn't receive the reagents."*<br><br>A medical officer describing shortage of reagents in the apex laboratories, said<br><br>*"Yes, like XXX (apex laboratory) didn't have reagents for creatinine for some time, so we couldn't send creatinine to XXX during that. You know when we talk about reagents there were lapses on and off"*<br><br>A programme manager explaining shortage of consumables, said,<br><br>*"Sometimes the need for glucose and cholesterol strips exceeds the routine annual requirement because of PSSP needs. So, the amounts provided by Regional medical supply division (RMSD) will become inadequate."*
Investigations at private laboratories with out-of-pocket expenditure	The HCWs complain that the patients are made to get their blood investigations done in the private laboratories due to non-availability of investigations through PMCI (not even with sample collection and transportation). The patient has to make out-of-pocket payments at private laboratories. The investigations in the private laboratories are	

	unaffordable for most patients. This leads to inequity and also there are concerns about quality of service.	
Unaffordability	The HCWs feel that the investigations in the private facilities are costly and are unaffordable for the majority of the patients seeking care in the PMCIs.	A medical officer describing the issue with investigations at private laboratories, said<br>*"We have to do investigations annually. The investigations for the patients would be as Lipid profiles, HBA 1C, Urine Nitrogen etc. These tests cost around 4000 to 5000 LKR. Actually, the people here are the parents who depend on their children. They are labourers. They are unable to spend 4000 to 5000 LKR for these tests. They are unable to get 3 meals a day"*
Inequity	The HCWs also feel that when the investigations are to be done from the private laboratories, only those who can afford to get them done actually avail themselves of this resource leading to inequity in access to laboratory services.	A medical officer said,<br>*"There are people who can afford and who get it done, and there are people who can't as well."*
Lack of Quality	The HCWs in the PMCIs mentioned that they don't have trust in the quality of the investigations done in the private laboratories. They feel most of the laboratories lack quality.	A medical officer said<br>*"And even the quality is not good in these. Although they do it in the private sector, spending their own money, still, we can't be assured that the quality is good in these reports."*<br>Another medical officer describing an event related to quality of investigations at the private laboratories, said<br>*"A patient who has money has given the blood samples to 3 different private places and all three reports differed from one another."*

Supplementary Table S4: Qualitative data on challenges in using Health Management Information System (HMIS) established in PMCI supported by PSSP in Sri Lanka, 2021

Sub-theme/ Category	Description	Quote
Personnel related issues	The challenges in availability of trained human resource to collect and enter data in the PMCIs as mentioned by the HCWs are described below.	
Shortage of data entry operators	The HCWs across the cadre complained that there is a huge shortage of data entry operators in the PMCIs, requiring either the nursing officer or the doctor to enter data in the HMIS. Also, HCWs feel that recruiting the data entry operators on a temporary basis is not helping as they quit the jobs when they get permanent job opportunities.	A programme manager describing the situation said, *"Very recently we appointed a DO (data entry operator) but unfortunately they get other appointments and leave… Then the problem arises again."*
Unaware of utility of HMIS	The HCWs does not appreciate the use of collecting and entering data in the HMIS, making them feel that data entry is an additional work thrust on them and without any use in their routine patient care. There seems to be lacunae in communicating the purpose of digitization during the training sessions.	A nursing officer managing data entry in the absence of the data entry operator, said *"Although we enter, there is no point because the same details are there in the books. We enter the same details with the same reference number, but these details cannot be searched to obtain details to the PSSP."*
Reluctance to change attitude	The programme managers feel there is resistance from the peripheral health staff to acclimatise to the need for systematically collecting and digitizing data. The programme managers complain that even on providing the required equipment, the peripheral health workers do not perform.	A programme manager describing the challenges with making people adhere to requirements of HMIS, said *"But, only the 'supply' is not enough, an attitudinal change also should be made, I have seen once a tab is provided, it is used by children at home to play games. People should understand this attitudinally, this is for work, for the ease of our work, even the empanelment can be done on the way without waiting till people come to hospital"*

Cumbersome process	The deficiencies perceived by the HCWs in the process followed in the PMCIs for collection and digitizing the data is detailed below.	
Duplication of effort- Paper based to electronic	The programme managers mention that some of the PMCIs maintain the registration register in which the demographic details of those registered are documented. The programme managers feel that HCWs might not enter these data into the portal in a timely manner. However, there is no standard procedure and the paper-based register is used for registration in some of the PMCIs.	A programme manager highlighting the issue said, *"There is a problem in the process of registration. Most are first entering in paper and it remains in writing mode as it is double work"*
Multiple formats for recording and reporting	The other issue highlighted by the HCWs is the confusion they have with multiple records and reporting formats issued by the various units. The NCD unit is concerned with the 'number of screening events' whereas the PSSP is looking for 'the proportion of empanelled people who are screened for NCDs'. There are multiple recording formats and the HCWs are not sure about the purpose of different registers and the indicators to be deduced from each one (this was evident during the quantitative data collection as the HCWs were not sure of data source for several indicators we tried to deduce).	A medical officer mentioning about multiple reporting formats, said *"Some have two PHN numbers when we checked the registry. These are problems too. Now only we know that, HLC and PSSP should be taken as two separate things. But previously we had analysed the related data together. Now they asked us to send the data of patients who don't have NCDs to the NCD unit. Data of patients who have screened for NCDs (if they have or don't have NCDs) are sent to the PSSP. The data of patients who don't have NCDs should be sent to the HLC. As previously we have recorded all the data together, now we have to exchange the data from one register to the other."*
Patients to carry the PHR for data entry	The HCWs complain that in spite of issuing the PHR, the patients fail to get it while making the follow-up visits to PMCI. Without patients getting the PHR, it is not possible to document in the PHR and also update the follow-up visit details in the HMIS. There is no paper based duplicate of	A medical officer talking about the issue, said *"We are issuing the PHR, but no one is using that. They can use it to write the feedback of the referral."*

	the PHR maintained in hospital and they depend only on the HMIS (which is yet to be established). This defeats the purpose of issuing the PHR.	
Delay in generation of PHN	The HCWs feel that due to the process of paper-based registration and later entry into the HMIS portal, there is a delay in the issue of the PHN.	A medical officer said,<br>*"Still we have to issue PHN numbers for some registered people. Because we don't have a permanent DO. That's the problem with delaying of issuing of PHN numbers."*
Issues with HMIS platform	Below are the issues that the HCWs highlighted related to the online HMIS module used for entering data in the PMCI.	
Online platform which crashes frequently	The HCWs mentioned that the online HMIS platform crashes frequently and will not be available for use when required. Any use of the HMIS module requires logging in to the portal.	A data entry operator said,<br>*"And like when I can't log in and create a PHN from the laptop, what I do is I try with my mobile. If I still can't create the PHN what we do is, we document all the details and ask the patient to come back and get the PHN (at the next visit)."*<br>A programme manager mentioning about need for offline version of HMIS said,<br>*"I have spoken to the company about offline registration facility, but they didn't agree for that. That is the major problem we are facing."*
Low server space for HMIS	On discussion with the HMIS programme managers, they too acknowledge such deficiency in the performance of the portal and mention that it is largely due to lack of domain space. Initially it was planned to host HMIS in a server with the capabilities of about 16 CPUs and 64 GB RAM.	

	However, as the HMIS is hosted in a government cloud, there are limitations and it was allotted only 16 GB RAM. With the low server space, the portal crashed when multiple people used it simultaneously. However, the government is planning to purchase new servers and the government has assured the HMIS programme managers that they will have better infrastructure so that the performance issue will be sorted out. The staff feel there is improvement, with breakdowns once a day in the initial period to 2-3 breakdowns a month in recent times.	
Lack of interactive dashboard	The HCWs feel there is no dashboard to display the performance and summary reports of data entered in the portal. There is no information available to the end-user on the data that they have entered. Also, the achievement status of the other PMCIs is not available to promote healthy competition to better the performance between the facilities.	A programme manager said, *"… I didn't find those as friendly. Actually, in number of times I discussed and tried to get access. But I couldn't. And, I strongly recommend, in most of the succeeded activities we implemented, we identified who is the first, who is the second, third etc. For example, we have 23 MOHs and when we displayed the first, second place in front of all, it highly motivated all"*
Difficulty in tracking of PHN	The HCWs involved in data entry mention that it is difficult to trace the patient file for updating the follow-up visit details by searching with the PHN number. Sometimes patients do not get the PHR and will not remember the PHN, in such cases it is difficult to track the individual details. Also, there may be transcription errors while entering the alphanumeric PHN of 10 characters and this can make it difficult to track patient details.	A nursing officer describing the challenges in tracking the patient details on the HMIS, said *"We enter the same details with the same reference number, but these details cannot be searched to obtain details to the empanelment."*

Infrastructural issues	The challenges perceived by the HCWs on the availability of the functional infrastructure for collecting and entering the data efficiently in the PMCI.	
Non-availability of printer and laptop	The HCWs at PMCIs complain that there is an inadequate supply of instruments like barcode, printer and laptop. Under the project, it was planned to issue the barcode printer to generate barcodes for patient health records (PHR) of each patient empanelled to improve the efficiency of tracking the patient form and updating the follow-up visit details. Without a barcode printer and reader, the data entry operator has to manually enter the PHN to search the patient details, which is prone to errors and not efficient. The HCWs also complain that one laptop may not be sufficient, when the empanelment is done simultaneously at PMCI and outreach clinics and also as back-up in case of breakdown of the laptop.	A nursing officer involved in data entry, said<br>*"We also need a barcode printer and another laptop. We only have one laptop and if it is break down we will be in a trouble."*
Lack of internet connectivity	As part of empanelment, the demographic details of individuals have to be entered in the online HMIS portal and generate a patient health number (PHN) to complete the registration. The HCWs complained that, with poor internet connectivity in the peripheral PMCIs, it is not possible to use the online HMIS portal for registration. Though the internet facility is made available in the facility, the connection is poor.	A programme manager mentioning the challenges with internet connectivity, said<br>*"They are facing problems regarding the connectivity and mobile data problems… But in some particular places like MONCD room and HLC, the internet doesn't function. When they do outreach clinics, they don't have the internet facility. So, they may need mobile phone/data as they can't do the registration without internet."*
Non-availability of PHR books	Once the PHN is generated, the individual is supposed to be issued with the PHR booklet for documenting the medical details in the future follow-up visits. However, the	A programme manager when asked about the extent of disbursement of PHRs, said

	HCWs complain that the PHRs were not readily available during the registration and were not able to provide PHRs to all the individuals at registration.	*"For the last two years, in most of the hospitals, they have given just a paper as PHR. So, for the moment we can't make any progress with that."*
